# Quantum Dot Labelling of Tepary Bean (*Phaseolus acutifolius*) Lectins by Microfluidics

**DOI:** 10.3390/molecules25051041

**Published:** 2020-02-26

**Authors:** Ricardo Cervantes-Jiménez, Lino Sánchez-Segura, Laura Elena Estrada-Martínez, Antonio Topete-Camacho, Elizabeth Mendiola-Olaya, Abraham Noé Rosas-Escareño, Carlos Saldaña-Gutiérrez, Mónica Eugenia Figueroa-Cabañas, José Luis Dena-Beltrán, Aarón Kuri-García, Alejandro Blanco-Labra, Teresa García-Gasca

**Affiliations:** 1 Facultad de Ciencias Naturales, Universidad Autónoma de Querétaro, Av. de las Ciencias s/n, Juriquilla, Querétaro CP 76230, Mexico; ricardocervantesjimenez@gmail.com (R.C.-J.); ln.laura.estrada.mar@gmail.com (L.E.E.-M.); carlos.saldana@uaq.mx (C.S.-G.); mofigueroaca@gmail.com (M.E.F.-C.); JLDena_271994@hotmail.com (J.L.D.-B.); aakuri@gmail.com (A.K.-G.); 2 Departamento de Ingeniería Genética, Centro de Investigación y de Estudios Avanzados del Instituto Politécnico Nacional, Unidad Irapuato, Guanajuato CP 36821, Mexico; lino.sanchez@cinvestav.mx; 3 Departamento de Fisiología, Centro de Ciencias de la Salud, Universidad de Guadalajara, Guadalajara CP 44340, Mexico; topete.antonio@gmail.com (A.T.-C.); a.rosas-escareno@outlook.com (A.N.R.-E.); 4 Departamento de Biotecnología y Bioquímica, Centro de Investigación y de Estudios Avanzados del Instituto Politécnico Nacional, Unidad Irapuato, Guanajuato CP 36821, Mexico; elizabeth.mendiola@gmail.com

**Keywords:** plant lectins, protein labelling, quantum dots, Tepary bean, microfluidics

## Abstract

Lectins are bioactive proteins with the ability to recognize cell membrane carbohydrates in a specific way. Diverse plant lectins have shown diagnostic and therapeutic potential against cancer, and their cytotoxicity against transformed cells is mediated through the induction of apoptosis. Previous works have determined the cytotoxic activity of a Tepary bean (*Phaseolus acutifolius*) lectin fraction (TBLF) and its anti-tumorigenic effect on colon cancer. In this work, lectins from the TBLF were additionally purified by ionic-exchange chromatography. Two peaks with agglutination activity were obtained: one of them was named TBL-IE2 and showed a single protein band in two-dimensional electrophoresis; this one was thus selected for coupling to quantum dot (QD) nanoparticles by microfluidics (TBL-IE2-QD). The microfluidic method led to low sample usage, and resulted in homogeneous complexes, whose visualization was achieved using multiphoton and transmission electron microscopy. The average particle size (380 nm) and the average zeta potential (−18.51 mV) were determined. The cytotoxicity of the TBL-IE2 and TBL-IE2-QD was assayed on HT-29 colon cancer cells, showing no differences between them (*p* ≤ 0.05), where the LC_50_ values were 1.0 × 10^−3^ and 1.7 × 10^−3^ mg/mL, respectively. The microfluidic technique allowed control of the coupling between the QD and the protein, substantially improving the labelling process, providing a rapid and efficient method that enabled the traceability of lectins. Future studies will focus on the potential use of the QD-labelled lectin to recognize tumor tissues.

## 1. Introduction

Lectins are a heterogeneous group of glycoproteins of non-immune origin, ubiquitous in nature but especially abundant in plants, which specifically and reversibly bind to carbohydrates, producing cell agglutination due to a non-catalytic domain in their structure [1,2,3,4,5]. These traits make lectins of great interest in the biomedical area, in which they have stood out for their effects against cancer cells [6,7,8]. The anticancer mechanisms of lectins are due to their binding to specific carbohydrates of cancer cell membranes, such as the sialyl-Lewis^x^ (SLe^x^), Thomsen-nouvelle (Tn), and sialyl-Tn (sTn) antigens [9]. This binding allows the detection of malignant cells [10,11] and, additionally, triggers cytotoxic activity through apoptosis and autophagy induction, resulting in the inhibition of tumour growth [2,12,13,14]. Although the complete mechanism of lectin activation has not been described, some signalling pathways have been proposed [4,15,16,17], and the cytotoxic effects of different lectins on cancer cells have also been reported [4,14,15,18,19,20,21].

Tepary bean (*Phaseolus acutifolius*) lectins have been studied because of their toxic and cytotoxic effects on cancer cell lines [22,23]. A Tepary bean lectin-rich fraction (TBLF), obtained by molecular size exclusion chromatography of the seeds’ extract, was tested on different cancer cell lines; where colon cancer cells showed the highest sensitivity to the treatment [22], related to apoptosis induction and cell cycle arrest [24]. Acute and subchronic assays of the TBLF—administered intragastrically to rats—exhibited low toxicity and good tolerability, as well as immune system activation. Adverse effects were related to the atrophy of the small intestine villi and colonic cryptic foci, hypertrophy of the pancreatic acini, and a decrease of body weight gain [25,26]. In preclinical studies, colon cancer was induced to rats using dimethylhydrazine or azoxymethane; where TBLF inhibited early premalignant lesions and aberrant cryptic foci, and cell death was related to caspase-dependent apoptosis [12]. The molecular structure of a Tepary bean lectin was elucidated by Torres-Arteaga et al. [27]. In order to study the specific interaction between the lectin and cancer cells, it is necessary to develop some novel imaging techniques for lectin detection.

The characterization of the interaction of lectins with cellular structures is fundamental for the understanding of their mechanisms of action and the subsequent biomedical uses [28]. However, the study of lectins demands high purity of the protein, and observation of the interaction with cellular components, as well as the intra and intercellular dynamics, requires the implementation of labelling techniques that facilitate their visualization and traceability.

Quantum dots (QD) are semiconductor nanocrystals used in fluorescence and confocal microscopy that have shown superior optical properties to those of conventional fluorescent dyes and proteins [29]. Most of these nanoparticles are biocompatible with proteins and antibodies, preserve a photostable emission during prolonged excitation, and show a tuneable range of excitation, a narrow emission spectrum and high fluorescence performance [30,31,32,33]; furthermore, they present a large surface area that enables the controlled conjugation of biomolecules [34]. All these characteristics are desirable for the labelling and monitoring of proteins in vitro and in vivo [35,36,37,38]. Considering their optical attributes, QD may also constitute a suitable option for imaging diagnoses of diseases [39].

On the other hand, microfluidics is a platform with biomedical applications that involves the performance, precise control, and manipulation of fluids and particles on the scale of tens to hundreds of micrometres, by the use of fluidic channels which allow the coupling or separation of particles. The advantages of using these devices include reduced volumes of reagents and samples, fast processing, ultra-high sensitivity, high portability, low cost and the alternative of automation [40,41]. Microfluidics also enable the integration of optical biosensors [42].

Different lectins have been conjugated with QD by distinct techniques for in vitro imaging [32,43,44,45]; however, to the best of our knowledge, microfluidics have not been used for lectin labelling. Therefore, the objective of the present work was to purify a Tepary bean lectin (*Phaseolus acutifolius*), couple it to quantum dot nanoparticles via microfluidics, and characterize the complex, with the aim of using it for lectin tracking on biological systems.

## 2. Results

### 2.1. Lectin Purification

**Figure 1:**
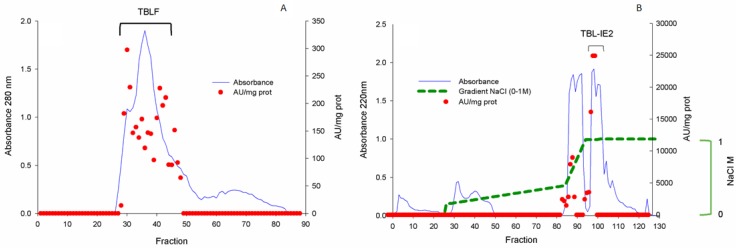
Chromatographic separation of the TBL-IE2 fraction. (**A**) Molecular size exclusion chromatography (Sephadex G-75). Red dots indicate fractions with agglutination activity (units per mg of protein), and the blue line shows the absorbance of the protein at 280 nm. TBLF is shown at the top. (**B**) Ion-exchange chromatography. Red dots indicate the fractions’ agglutination units per mg of protein, the absorbance at 220 nm of the protein fractions is shown in blue, and the green line marks the gradient from 0 to 1 M NaCl. The second peak, named TBLF-IE2, is indicated at the top.

**Figure 2:**
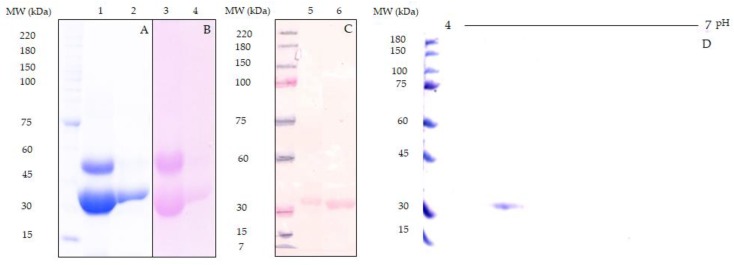
SDS-PAGE profile for the TBL-IE2 fraction. A 10% SDS-PAGE was performed. (**A**) Coomassie staining for TBLF and TBL-IE2 electrophoresis; (**B**) Schiff’s periodic acid staining of TBLF and TBL-IE2; (**C**) Western Blot for TBLF and TBL-IE2; (**D**) two-dimensional electrophoretic profile for TBL-IE2.

A Tepary bean lectin fraction (TBLF) was purified by molecular size exclusion chromatography; protein was detected at 280 nm and fractions with agglutination activity were pooled (12,300 Agglutination units (AU)/protein mg) and subsequently separated by ionic-exchange chromatography, where two main peaks with agglutination activity were observed, and the second peak (TBL-IE2) was selected (Figure 1). Figure 2 shows the SDS-PAGE analysis for the TBLF and the TBL-IE2 fractions. Following the Schiff/periodic acid stain, two protein bands were found for TBLF, with approximate molecular weights of 28 and 56 kDa while a single protein band for TBL-IE2 was observed, with an apparent molecular weight of 28 kDa. This protein band was recognized by Western blot analysis using the specific antibody for Tepary bean lectin. The two-dimensional electrophoresis for the TBL-IE2 fraction showed the same molecular weight protein with an isoelectric point of approximately 4.7.

### 2.2. Lectin Labelling and Analysis

**Figure 3:**
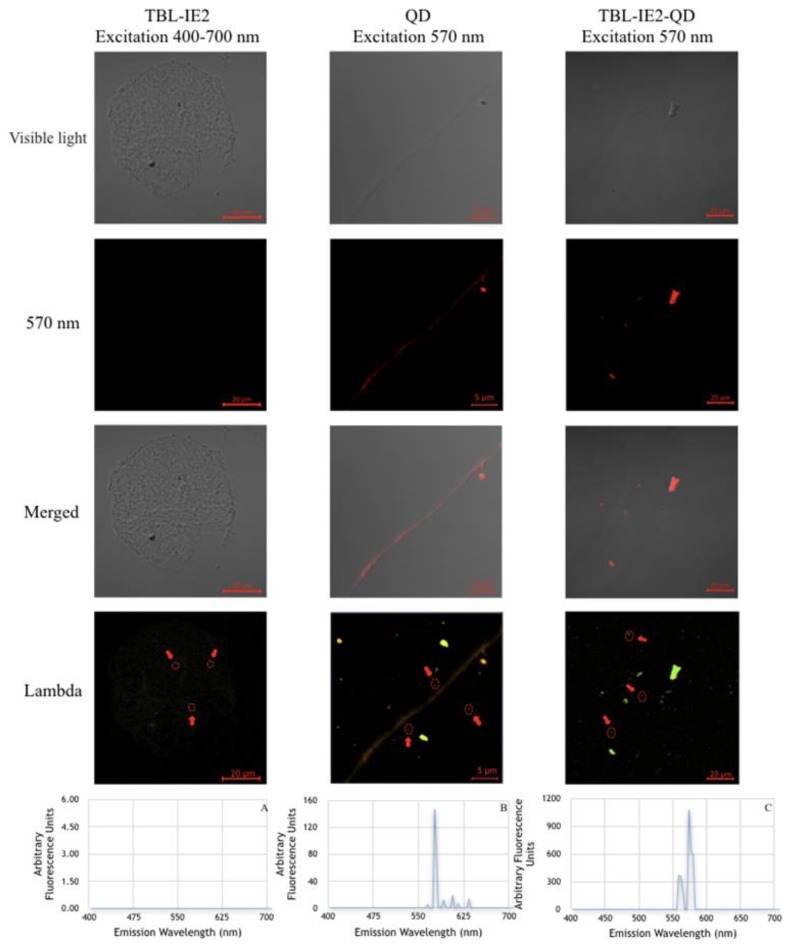
Fluorescence images for the TBL-IE2-QD complex. Analysis in visible light, excitation analysis at 570 nm, visible light and excitation at 570 nm merged, and lambda analysis from 400 to 700 nm (taking a reading every 2 nm). (**A**) TBL-IE2, lambda figure size 94.01 µm × 94.01 µm where no fluorescence was observed; (**B**) Quantum dots, lambda figure size 34.46 µm × 34.46 µm, with a single peak at 570 nm; (**C**) Analysis of the TBL-IE2-QD complex, lambda figure size 134.95 µm × 134.95 µm—a peak can be observed at 574 nm, and a second peak at 560 nm with less intensity.

**Figure 4:**
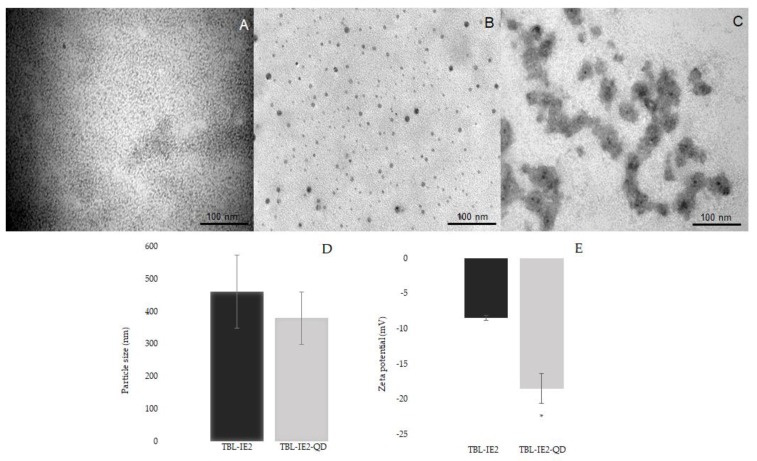
Physicochemical characterization of TBL-IE2 and TBL-IE2-QD complex. Transmission electron microscopy images for the TBL-IE2 and TBL-IE2-QD. The differences between the unlabelled and labelled lectin are shown at 180,000 Kv magnification; (**A**) TBL-IE2, (**B**) TBL-IE2-QD complex coupled by microfluidics, and (**C**) TBL-IE2-QD complex coupled by covalent bonding. (**D**) Particle size analysis of TBL-IE2, compared to independent assays of the TBL-IE2-QD conjugate. (**E**) Zeta potential analysis of TBL-IE2, compared to independent assays of the TBL-IE2-QD conjugate. Asterisk shows statistically significant differences (*p* < 0.05).

After the purification, the lectin was successfully labelled with QD (TBL-IE2-QD); 75% of the agglutination activity was conserved (9300 AU/mg of protein). The purified protein did not exhibit autofluorescence at the 400–700 nm spectrum (Figure 3). On the other hand, the analysis of QD alone showed one peak of fluorescence at 570 nm, and the TBL-IE2-QD complex showed a spectral emission between 560 nm and 574 nm. The results for the transmission electron microscopy showed different molecular sizes for TBL-IE2 and TBL-IE2-QD, as shown in Figure 4. The particle size was not significantly different between TBL-IE2 and TBL-IE2-QD, with averages of 461 and 379 nm particle diameter, respectively. The zeta potential for TBL-IE2 was −8.23 mV, and for the TBL-IE2-QD complex was −18.51 mV. It was possible to observe that, when using a non-microfluidic method, the complexes formed heterogeneous clusters.

### 2.3. Cytotoxic Effects of TBL-IE2 and TBL-IE2-QD on HT-29 Cell Line

**Figure 5:**
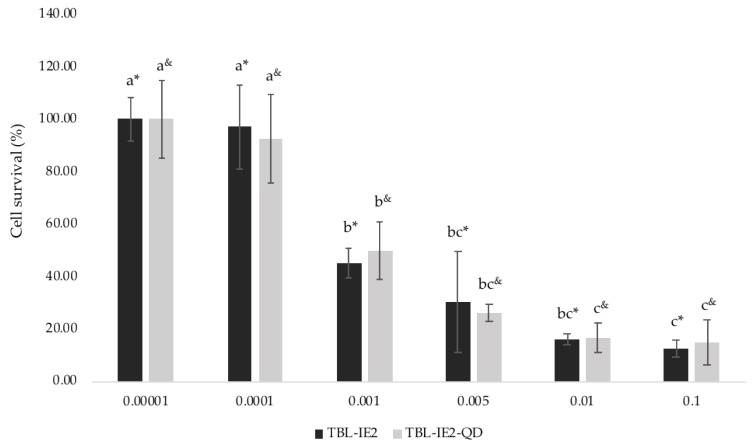
Cytotoxic effect of TBL-IE2 and TBL-IE2-QD complex on HT-29 cells. The cell survival percentage rate after different treatments of TBL-IE2 and TBL-IE2-QD is shown. Small letters (*) TBL-IE2 and (^&^) TBL-IE2-QD indicate statistically significant differences between concentrations for each treatment (Tukey, *p* < 0.05).

The cytotoxic effect of both the labelled and unlabelled lectins was observed on HT-29 human colon cancer cells (Figure 5). A similar effect was observed between them, since the TBL-IE2 showed a CL_50_ of 1.0 × 10^−3^ mg/mL and TBL-IE2-QD, 1.7 × 10^−3^ mg/mL.

## 3. Discussion

There is much in the literature about the purification processes of lectins; however, their conjugation with nanoparticles for microscopic visualization has only been explored in the last 15 years. At least a dozen lectins have been coupled with cadmium-telluride (CdTe), sulfide (CdS), and selenide (CdSe) QD, for various purposes as biosensors [46,47], multimodal nanoprobes [48], theranostic systems [49], and for the study of cancer glycobiology [50].

Different methods for lectin–QD coupling have been developed by distinct research groups, including adsorption, electrostatic, and hydrophobic interactions; covalent bonding, which entails the formation of an amide, imine, or disulphide bond; stabilizer exchange, where a thiol-containing molecule is added to the QD; and QD surface modification with NH_2_ polyethylene glycol [32]. Some labelling techniques report the recognition of carbohydrates in fungi, bacteria, or cancer cells using traditional methods. It has also been found that labelling is improved using coupling agents such as EDC or sulfo-NHS [51,52,53], although the physicochemical characteristics of the lectin–QD complex have not been fully described. To date, no studies have reported the coupling of a lectin with QD using a microfluidic platform.

The selection of the conjugation process plays an important role, as the biochemical properties of the lectin and the optical qualities of the QD need to be preserved [32]. In the present work, after labelling, the properties of both the lectin (agglutination and cytotoxicity) and the QD (fluorescence emission) were conserved. Some other methodologies have been reported for the purification of lectins from other sources [54]; however, one of the main problems of these methods is the poor yield of the protein of interest. Therefore, it is important to focus on the production of recombinant lectins [55].

Our results showed that the purified lectin TBL-IE2 exhibited an apparent molecular weight of 28 kDa, similar to that reported by Garcia-Gasca et al. [22], that was determined by protein and glycoprotein staining and by Western blotting. The two-dimensional electrophoresis showed a single protein band, with an isoelectric point of approximately 4.7, as reported by Torres-Arteaga et al. [27], slightly lower than that reported for Con A lectin, which was determined to have an isoelectric point of 5 [56,57]. After the labelling, the TBL-IE2-QD agglutination activity was conserved at 75%, as already reported by other authors [58].

The lectin did not show autofluorescence, since lectins that present saccharides are non-fluorescent molecules [59]. On the other hand, the analysis of the QD alone showed one peak of fluorescence spectral emission at 570 nm. This emission was similar to the fluorescence reported for cadmium telluride QD for protein-labelling processes [60]. The spectral emission of the TBL-IE2-QD complex was similar to the pure QD, suggesting that the interaction between carboxyl and the amino functional groups of the QD surface with lectin amino acids did not produce significant changes in the fluorescence emission.

By TEM analysis, morphological differences were observed between the labelled and the unlabelled lectins, where it was possible to observe QD bound to the lectin, since free QD were previously eliminated by dialysis. The microfluidic method has been utilized in previous works [61] for the coupling of different particles, but—to the best of our knowledge—no works have reported the labelling of lectins with QD by this technique.

Previous works from other authors concerning lectin labelling with QD by non-microfluidic methods have been reported, although the physicochemical characterization of the obtained complexes has been only reported rarely [62]. In the present work, a visual TEM comparison of the morphology of the lectin–QD complexes obtained by microfluidics versus covalent bonding technique was performed, where heterogeneous complexes with QD clusters were observed when covalent labelling was used. With the use of the microfluidic method, the QD were homogeneously distributed among proteins, an observable trait in all samples, indicating the reproducibility of the technique.

On the other hand, an advantage of the use of the microfluidic technique was the low usage of samples; for example, Carvalho et al. [58] reported using 28 mg/mL of their lectin, while we used 20 μg/mL. The particle size was determined for both the unlabelled and labelled lectins, where no significant differences were observed (461 and 379 nm, respectively). The complex decreased 18% in size, suggesting that the lectin-QD conjugation compacted the protein structure. The zeta potential of TBL-IE2-QD was significantly lower than the one of TBL-IE2, which may be attributed to the QD net charge. It has been reported in previous studies that the most appropriate pH for the labelling of several lectins is 7 [32,45], when labelling is achieved by adsorption or covalent bonding with glutaraldehyde [63,64]; however, in the present work, the best results were obtained at pH 6.

Regarding the cytotoxic effect, a similar dose-dependent effect was observed, where the LC_50_ values for the TBL-IE2 and the TBL-IE2-QD conjugate were 1.0 × 10^−3^ and 1.7 × 10^−3^ mg/mL, respectively, indicating that the labelled lectin retained its cytotoxic activity. This result suggests that the lectin-QD interaction did not affect the biological activity of the lectin, thus allowing its use for the study of the mechanism of action. However, the toxicity of QD remains a concern in biological systems, because of the release of heavy metal ions (as Cd^+2^), the generation of reactive oxygen species, and intracellular effects [65,66]. Nevertheless, the cytotoxicity of QD depends on the fabrication materials, coating and other factors, so their harmfulness cannot be generalized. After the labelling, the cytotoxic effect of TBL-IE2 on HT-29 cells remained unchanged. The available reported results suggested that protein labelling with QD represents a promising tool for the development of diagnostic methods such as cancer marker detectors [67], particularly through the use of lectins [68].

## 4. Materials and Methods

### 4.1. Biological Materials and Quantum Dots

Tepary bean seeds were purchased in a local market at Hermosillo, Sonora, Mexico, and a sample was identified and deposited in the Dr. Jerzy Rzedowski Herbarium at the Faculty of Natural Sciences, Autonomous University of Queretaro, Mexico (QMEX00007888). Cadmium-Telluride Quantum Dots (CdTe QD) functionalized with carboxyl groups (COOH-) were purchased as solid powder nanoparticles (4–6 nm) (Sigma-Aldrich^®^, St. Louis, MO, USA). Human HT-29 colorectal adenocarcinoma cell line was obtained from ATCC^®^ (HTB-38™) (American Type Culture Collection Rockville, CT, USA).

### 4.2. Lectin Extraction and Purification

Lectins from Tepary bean seeds were extracted using approximately 100 g of defatted raw bean flour dissolved in 1 L deionized water [22]. Briefly, the crude extract was precipitated from 40% to 70% ammonium sulphate saturation, and the precipitated proteins were collected after centrifugation at 39,200 ×*g* for 30 min. The pellet (P40–70) was dissolved in 15 mL of deionized water and dialyzed in a 3 kDa membrane (Spectrum Laboratory, Inc. Standard RC Tubing No. 9200676) against deionized distilled water at 4 °C, until it reached a 2 μΩ conductance. The protein obtained from the sequential precipitation was dialyzed and then fractionated using a Sephadex G-75 gel filtration chromatography column (155.5 × 1.55 cm) equilibrated with 0.02 M ammonium bicarbonate buffer, with a pH of 7.8 at 4 °C, collecting 3 mL samples at a flow rate of 0.3 mL/min. The protein was monitored at 280 nm and agglutination activity was assayed using 2% glutaraldehyde-fixed [69] human A^+^ erythrocytes (provided by the Querétaro State Blood Transfusion Center), following the method described by Jaffé [70] and Adamová et al. [71] with modifications.

The fractions with agglutination activity were pooled and separated by ionic exchange chromatography using an Econo Pac High Q Cartridge, 1 × 5 cm (Bio Rad), equilibrated in 0.01 M Tris-HCl buffer (pH 8). The adsorbed protein was eluted with a linear gradient from 0 to 1 M of NaCl in 0.01 M Tris-HCl pH 8. Fractions of 2 mL were collected at a flow rate of 1.0 mL/min and protein was determined at 220 nm. Fractions with agglutination activity were pooled, dialyzed, lyophilized, and evaluated using SDS-PAGE [72] and 2D electrophoresis. All samples were analysed by Bradford staining [73]. Samples that displayed a single protein band after SDS-PAGE, as well as a single band after carbohydrate staining, were selected for coupling to QD nanoparticles. All procedures were carried out at 4 °C.

### 4.3. One- and Two-Dimensional Electrophoretic Separations and Immunoblotting Determination

The purified lectins were separated by SDS-PAGE, using 10% resolving gels according to Laemmli [72]. Coomassie blue R-250 was used for protein staining. Glycoproteins present in the polyacrylamide gels were stained following the PASS technique [74,75]. In addition, the proteins were transferred to a nitrocellulose membrane. A primary anti-rabbit antibody for Tepary bean lectins previously designed in our laboratory was used. Subsequently, a secondary antibody (AffiniPure Anti-Rabbit IgG Santa Cruz, CA, USA) was employed for specific protein identification. The immunoreactive proteins were visualized using the Amersham^TM^ ECL^TM^ Western Blotting Analysis System^M^ kit. Two-dimensional electrophoresis was carried out according to Görg et al. [76] using a 7 cm immobilized pH gradient (IPG) strip with a linear pH gradient from pH 4 to 7. The second-dimension separation was carried out using a 10% polyacrylamide gel. Silver staining was performed according to the protocol reported by Blum et al. [77].

### 4.4. TBL-IE2-QD Coupling by Microfluidic Technique

The coupling procedure was carried out with a polydimethylsiloxane (PDMS) microchip with a channel diameter of 0.7 mm, manufactured and kindly provided by Dr. Natalia Hassan from Metropolitan Technological University of Chile (Santiago, Chile). First, the microchip was washed with a combination of H_2_O_2_:HCl:H_2_O, 1:1:5 (v/v/v) at a flow rate of 1.2 mL/h using two infusion syringe pumps (KDS 100 Legacy, KD Scientific, Holliston, MA, USA), followed by rinsing with 1 mL of milliQ water at a flow rate of 6 mL/h. Subsequently, 300 μL of trimethyl octadecyl silanol at 20 μL/mL was injected through the channels at a flow rate of 1.8 or 2.4 mL/h, and a sequential rinse was performed with 2 mL of dimethyl sulfoxide (DMSO), 2 mL of milliQ water, and 2 mL of acetone. Air was applied under pressure in order to dry the microchip and 15 cm long Tygon^®^ hoses were used and replaced after the preparation steps. Thereafter, a lectin solution was prepared by dissolving 200 μg of lectin in 10 mL of 0.1 M MES buffer pH 7.

**Figure 6:**
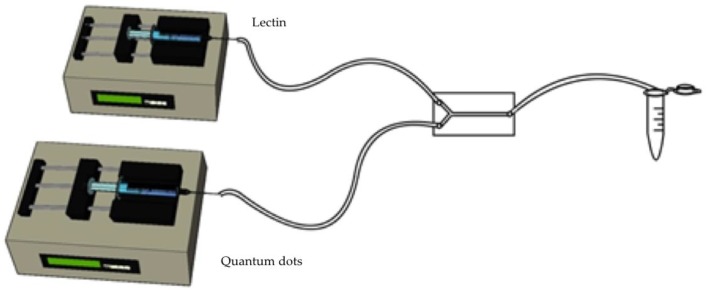
Diagram of the microfluidic labelling method. A lectin solution on one side, and a Quantum Dot solution on the other, both administered by infusion syringe pumps, merge in the polydimethylsiloxane (PDMS) microchip and then flow to a common pool.

QD stock solution was prepared by dissolving 10 mg of CdTe-QD powder in 1 mL of deionized water (pH 6). The QD were then diluted in 1× PBS (pH 6) at a concentration of 9.5 × 10^−3^ mg/mL. The lectin (20 μg/mL) was placed in a 10 mL syringe (Terumo^®^, NJ, USA), supplied at a flow rate of 1.8 mL/h, while QD were placed in a 10 mL a glass syringe (Hamilton^®^, Reno, NV, USA) to avoid interaction with the syringe surface, and supplied with a flow rate of 2.4 mL/h. The product was collected in low-protein-binding polypropylene microtubes (Figure 6) and dialyzed through a 3.5 kDa membrane to eliminate the uncoupled QD, after which the corresponding characterization tests were performed. In order to compare the efficiency of microfluidics, the same lectin and QD dilutions at the same pH were used for coupling by covalent bonding (a non-microfluidic method), using glutaraldehyde as a coupling agent, which were kept in continuous agitation for 15 min and then dialyzed.

### 4.5. Determination of the TBL-IE2, QD, and TBL-IE2-QD Fluorescence Emission by Multiphoton Microscopy

The TBL-IE2 lectin, the QD alone, and the TBL-IE2-QD complex obtained by microfluidics were analysed by laser multiphoton microscopy. Samples were independently mounted on glass slides and covered with high-performance Zeiss cover glasses (D = 0.17 mm ± 0.005 mm refractive index = 1.5255 ± 0.0015, Abbe number = 56 ± 2) and observed under a microscope (LSM 880 NLO, Zeiss, Germany) equipped with a multiphoton laser Ti: Sapphire (Chameleon vision II, COHERENT, Scotland, UK) capable of tuning between ranges from 690 to 1060 nm. The operating conditions in all experiments were Chameleon laser-operated at 1.0% power and with an open pinhole. The complete areas for observations were carried out with immersion oil objective 60×/1.3, NA ∞−0.17, Zeiss Plan Neofluar. Images were acquired by separating the emission into three channels, blue or UV region (371–440 nm), green/yellow region (488–550 nm), and red region (560–730 nm). For spectral detection, “lambda mode” was used by ZEN lite blue 2.5 software (Carl Zeiss Microscopy GmbH, Jena; Germany), scanning emission from 400 to 700 nm for all samples, taking a reading every 2 nm. The images were obtained severally by excitation in two wavelengths at 780 nm and 850 nm. All images were captured in CZI format at 1131 × 1131 pixels, version Zen Blue 2.5 2018. A lectin solution was prepared by dissolving 200 μg of lectin in 10 mL of 0.1 M MES buffer (pH 7).

### 4.6. Morphological Analysis by Transmission Electron Microscopy (TEM)

The morphology of the TBL-IE2-QD complex was examined with a Morgagni^TM^ 268 transmission electron microscope (Philips/FEI, Eindhoven, The Netherlands). For the morphology analysis, 3 μL of the sample was placed onto 200 mesh Cu and incubated for 10 min in presence of uranyl. Drying of the sample was carried out at room temperature for 5 min. The samples were then contrasted with 2.5% uranyl acetate (Electron Microscopy Science; Hatfield, PA, USA) and incubated for 15 min. The operating conditions in each of the experiments were 80 kV high voltage (EHT), captured in high magnification in TIFF format with a 1376 × 1032-pixel size, and captured in greyscale. In this format, 0 was assigned to black and 255 to white in the greyscale.

### 4.7. TBL-IE2-QD Zeta Potential and Hydrodynamic Diameter Determination

The hydrodynamic diameter (Z-average) and zeta potential of the TBL-IE2 and TBL-IE2-QD complex were determined with a Zetasizer ZS90 (Malvern^®^, Malvern, UK). Briefly, 20 μg/mL samples were placed in polystyrene disposable cells. Each sample was analysed on a Zetasizer Nano ZS90 DLS, at a 90° angle in triplicate, each measurement consisting of 10 runs of 60 s. For the zeta potential and particle size, the samples were placed in folded-capillary disposable cells and evaluated in triplicates of 15 runs. Results were reported as mean ± standard deviations.

### 4.8. Cytotoxicity Assay

A total of 1 × 10^4^ HT-29 cells were seeded in 24 well microplates (Corning^®^) (Sacramento, CA, USA) and filled with 0.5 mL of Dulbecco’s modified Eagle’s medium (DMEM) supplemented with 10% foetal bovine serum (FBS) (Biowest, Nuaillé, France). After 48 h, the medium was substituted with 1 mL DMEM with 2% FBS for cell cycle synchronization. After 24 h, the following treatments were applied: 0.005, 0.001, 0.05, 0.01, and 0.1 mg/mL of TBL-IE2 or TBL-IE2-QD dissolved in 1 mL Dulbecco’s modified Eagle’s medium (DMEM) with 2% bovine serum albumin (BSA). The control wells were filled with 1 mL DMEM with 2% BSA. Cells were trypsinized and collected after 8 h of treatment, and immediately counted using a Neubauer chamber. All processes were carried out in quadruplicate, in three independent assays.

### 4.9. Statistics

To determine whether the cytotoxic effects of TBL-IE2 lectin and the TBL-IE2-QD complex were significantly different, an analysis of covariance (ANCOVA) was performed using concentration as a covariate. Data analysis was performed using R software (The R Foundation for Statistical Computing, Vienna, Austria), version 3.5.3 [78].

## 5. Conclusions

Previous work with Tepary bean lectins has demonstrated the effectiveness of this proteins in triggering cytotoxic effects on malignant cell lines and tissues. In the present work, a lectin (TBL-IE2) from this bean was purified, characterized, and successfully coupled to quantum dot nanoparticles by microfluidics. This technique has the advantage of low sample usage and controlled coupling, and resulted in the formation of homogeneous complexes that enabled their optical visualization and physicochemical characterization. The TBL-IE2-QD retained 75% of its biological activity, showing a similar cytotoxic effect respective to the native lectin, and also maintained similar agglutination activity. Our results suggest that the lectin–QD complex may be suitable for use in the future for TBL-IE2 tracking and bioimaging for in vitro assays, in order to increase the knowledge about its interaction with cancer cells. Further experiments are needed to analyse its viability for use in in vivo systems.
